# Supplementary figures and images for: Human ABCB1 with an ABCB11-like degenerate nucleotide binding site maintains transport activity by avoiding nucleotide occlusion
Source: PLoS Genet. 2020 Oct 8;16(10):e1009016. doi: 10.1371/journal.pgen.1009016 (PMC7544095; doi:10.1371/journal.pgen.1009016)

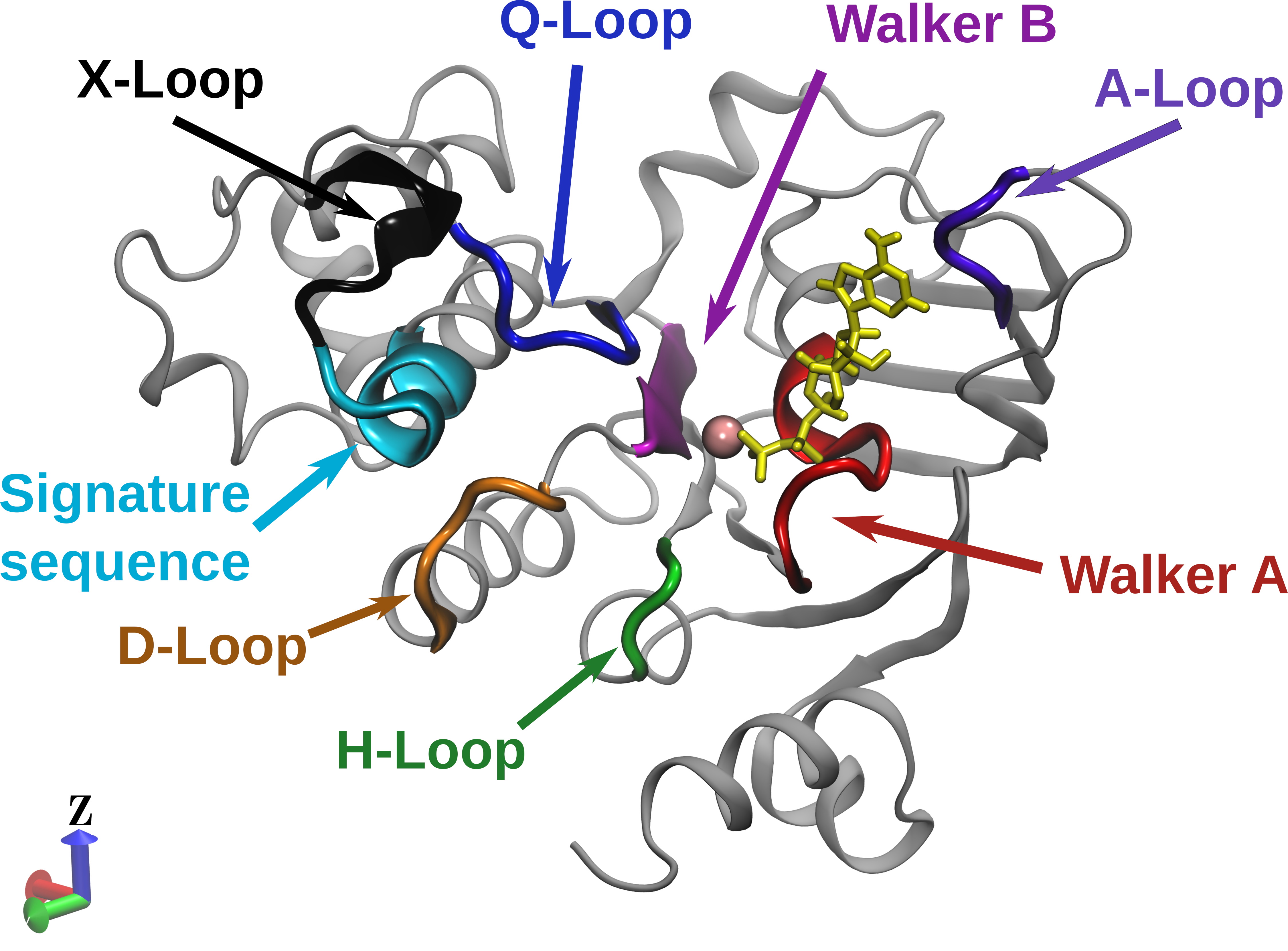

Supplement: S1 Fig — The known motifs are highlighted by color, ATP is show in yellow, the Mg2+ ions in pink. The arrows in the lower left corner indicates the orientation of ABCB1 relative to the membrane. The green and red arrows are oriented parallel to the membrane plane, the blue arrow indicates the direction perpendicular to the membrane. (TIF) [file pgen.1009016.s001.tif]

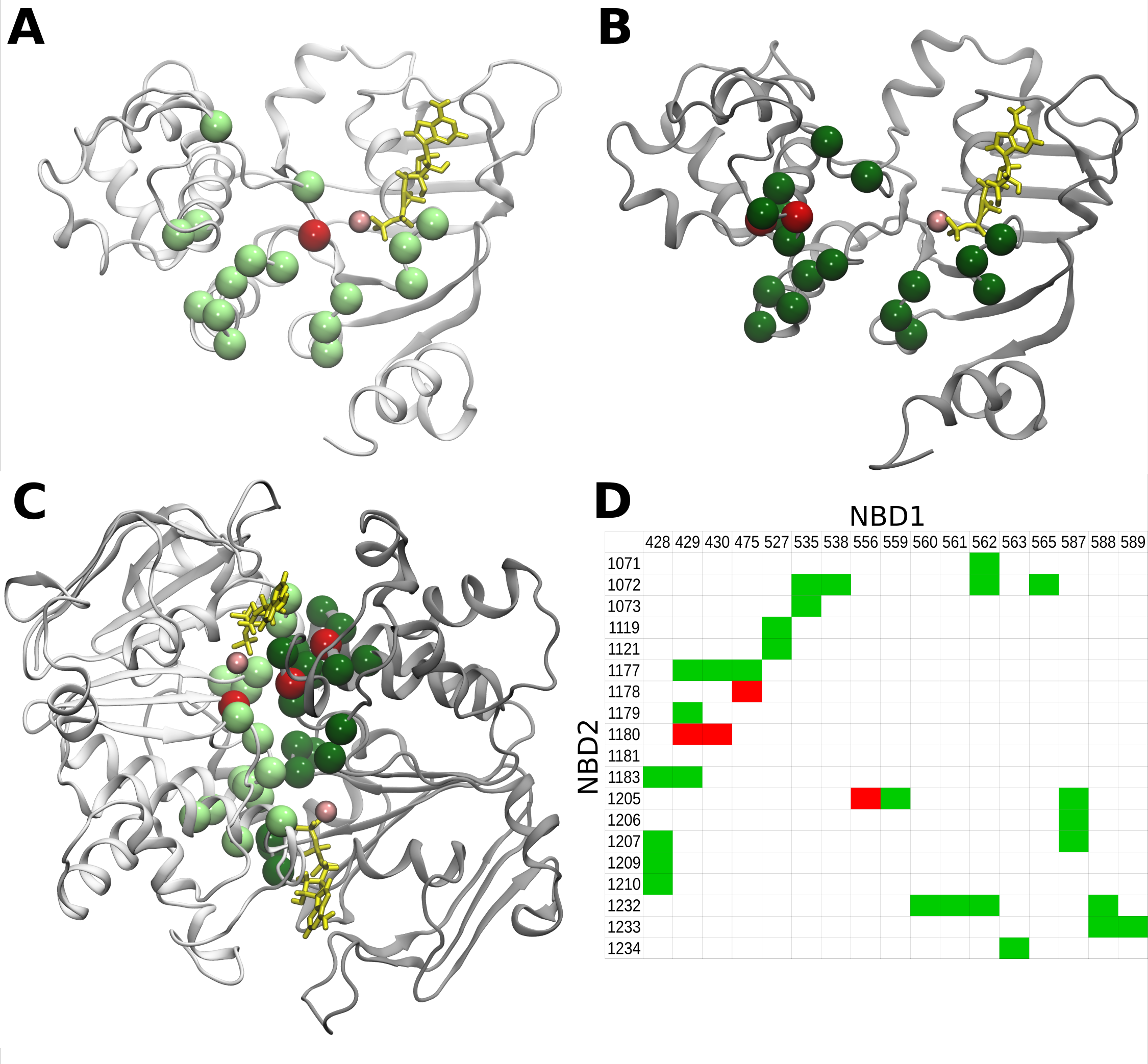

Supplement: S2 Fig — (A) Cartoon representation of NBD1 of ABCB1, oriented as in supporting S1 Fig. ATP is shown as yellow sticks, Mg2+ as a pink sphere. Residues in direct contact with NBD2 are indicated by light green sphere of their respective Cα atoms. Residue 556 is highlighted in red. (B) Cartoon representation of NBD2 of ABCB1, shown similar as NBD1 in panel A. Residues directly interacting with NBD1 are highlighted by dark green sphere of their respective Cα atoms. Residues 1178 and 1180 are indicated in red. (C) Top view of the NBD dimer showing the Cα atoms of all residues that are in direct contact. (D) The matrix summarize all contacts across the NBD-dimer interface, indicating in red the residues mutated in the quadruple mutant. ATP acts as glue [21, 67] and interaction hub, while shielding residues from direct interaction. (TIF) [file pgen.1009016.s002.tif]
