# Supplementary material for: Human ABCB1 with an ABCB11-like degenerate nucleotide binding site maintains transport activity by avoiding nucleotide occlusion
Source: PLoS Genet. 2020 Oct 8;16(10):e1009016. doi: 10.1371/journal.pgen.1009016 (PMC7544095; doi:10.1371/journal.pgen.1009016)
Supplement: Attachment — Submitted filename: Response-to-reviewer-comments_v7.doc [file pgen.1009016.s003.doc]

Response to the reviewers’ comments

*Reviewer #1: The manuscript by Goda, Donmez-Cakil, Chiba, Stockner and coworkers attempts to address a critical biological question regarding the nucleotide-dependent conformational changes needed to occur within the Nucleotide-binding domains (NBDs) and subsequently to be transmitted to the translocator (TMDs) for transport to be achieved. NBDs represent the most conserved domains of the largest group of transport proteins known (ABC proteins) that are found in all kingdoms of life, and are also present in transport unrelated ABC proteins. Numerous models have been proposed to address the means by which the nucleotide occupancy state (ATP, ADP, etc) and hydrolysis affects the conformational states of the NBDs and the cross-talk of the NBD-TMD with changes occurring at the TMDs (e.g. substrate binding to TMDs). It is crucial to address if there are common -universal- principles governing the function of all ABC proteins, or completely different models describe the function of different ABC proteins. To answer such questions the investigators compared 2 “flavors” of NBDs found in ABC exporters: Those that form 2 canonical Nucleotide binding sites (NBS) and those having one degenerate NBS. Wisely, to accomplish this, they selected 2 highly related proteins (ABCB1 and ABCB11, sequence identity:49%), as in such a case it is possible to address if it is achievable to “transform” one category to the other (by pinpointing the differences), what is needed for this conversion and the concomitant structural and functional repercussions. The NBS1 of ABCB1 has been transformed to the degenerate NBS1 of ABCB11 by introducing a mutation in the conserved E of Walker B motif and explored the structural and functional repercussions of this transformation. This transformation was proved to be deleterious, but the authors managed to create a gain of function derivative in which the NBD-NBD interface was “copied” to resemble that of ABCB11.*

*A combination of biochemical/functional experiments with dynamic simulations yielded important mechanistic insights that should be published. However, to evaluate publication in PLOS Genetics, a part of the conclusions needs to be reconsidered, as contradictory to published experimental data. Moreover, additional analysis is needed to reach the conclusions that would allow publication in PLOS Genetics. Finally, some figures and text should be adapted to render the conclusions clear and straightforward to the readers.*

We thank the reviewer for the positive assessment of the aim and design of our study. Please find below a point-by-point response to the comments.

*1. Fig.2: ABCB1 transporters (WT/E556M/Quadruple) are identically expressed and incorporated within the cell surface, a very important control experiment for the subsequent results. By comparing the conformation of ABCB1 in untreated, CsA-treated and ATP depleted cells the authors concluded that the E556M derivative obtains an ATP-induced trapped state. Since the assay reports on the translocator conformational state, it means very importantly that this conformational event, initiated at the NBDs, is also transmitted to the translocator domains. The trapped state can be completely removed by the Quadruple mutations.*

We thank the reviewer.

*2. Fig.3: The ATP-driven conformations are indeed dependent on ATP concentration, this is very clear from this figure. However, given the fact UIC2-binding is extremely low for the E556M derivative, even in untreated cells (Fig.2B, Untreated, middle column); how did the authors managed to obtain a dose response curve (as a function on MgATP concentration) for the E556M derivative? The authors should introduce a right Y axis in all Fig.3 panels with the absolute together with the relative (left Y axis) values of UIC2 binding.*

Low UIC2 binding of the E556M mutant is only observed in the presence of ATP (Figure 2B, “Untreated”). In the absence of ATP, binding of UIC2 to E556M is indistinguishable from that to wild-type (Figure 2B, “ATP depletion”). This result can be explained when we consider that under physiological conditions, the E556M mutant stably binds ATP, blocking the transporter in an outward facing conformation, which is not recognized by UIC2. However, in the ATP-depleted condition, in the absence of nucleotides, the transporter (wild-type and E556M alike) are in an inward facing conformation, which is recognized by UIC2. Thus, the starting points (ATP-depletion) of the dose-response curves shown in Figure 3 are the same, indicating equal labeling by UIC2. Figure 3 shows that an essentially identical full scale signal is found in the absence of ATP in wild type and mutants (also in absolute values, reflecting the fluorescence of the UIC2-labeled cells). At high ATP concentrations and in the presence of vanadate the signal is low. Thus, a comparable signal range is found in wild type, the E556M and the quadruple variant. To make this point clear, we now explicitly state this circumstance in the results section.

*3. Fig.4: I agree with the fact that in the quadruple mutant the substrate stimulated (which is the one meaningful for function) steady-state ATPase activity is restored, while completely abolished in the E556M derivative. The steady-state basal ATPase activity of the quadruple mutant is though approximately half of that of wild-type. Is that due to the fact that steady-state ATP hydrolysis occurs only in NBSII? I would like to insist on the term steady-state ATP hydrolysis instead of just ATP hydrolysis also for issues that I will detail afterwards.*

We agree with the Reviewer that our experiments measure steady state ATPase activity, and we corrected the text accordingly. The question raised by the reviewer is very intriguing, but notoriously difficult to answer. One possibility to explain the observed reduction of the basal steady-state ATPase activity by 50% in the quadruple mutant could be that only one NBS instead of two are capable of hydrolysis. This hypothesis would also imply that ABCB1 hydrolyses two ATP per transport cycle. However, at this point we cannot rule out several alternative explanations. The important finding for the argument developed in this manuscript is that ATPase activity is regained by introducing additional mutations in the single E556M variant, which does not hydrolyze ATP. The major conclusion of the manuscript, i.e. the three additional mutations allow the transporter to escape the locked state of the E556M mutant, is based on this change in quality despite the absence of the catalytic glutamate. Encouraged by the Reviewer’s question we now shortly discuss different interpretations of the quantitative changes in the steady-state ATPase activities.

*4. Fig.5A: Indeed, the restoration of the substrate stimulated steady-state ATPase activity (Fig.4) leads to restoration of transport. The fact that steady state ATPase activity comes likely only from NBSII in the quadruple derivative, this seems to lead to half transport activity. The authors should comment on that. If that is true, this implied that ABC exporters with one degenerate NBS, retain half on the activity with the same (half) ATP consumption (Fig.4). Fig.5B: In contrast, the results for Calcein-AM are not so clear. Here I would argue a very small difference between E556M and the quadruple derivative, and a huge difference between the quadruple derivative and wild type [especially if one would subtract the minus transporter values (first column)]. The authors should comment on that. Does a degenerate site increase transport selectivity?*

As indicated in response to comment 3, we agree that NBS2 may be the sole catalytic center of the quadruple mutant, but we cannot rule out alternative explanations. To address the reviewer’s second point, we have performed additional experiments with a broader range of Hoechst 33342 and calcein-AMconcentrations. The results are shown in a new figure (Figure 5), which reveals a two-fold difference in the transport activity factor between wild type and the quadruple mutant for both substrates. The concentration-dependent changes of the transport activity factor is comparable between wild type and the quadruple mutant. Therefore, presently we have no evidence to suggest that a degenerate site would change the selectivity of the transporter.

*5. Fig.6: The results indicate that NBS1 of the quadruple mutant is more “relaxed” than that of WT and the E556M derivative. This according to the authors is the reason of by-passing the ATP trapped state of E556M. This would mean that the dynamics of canonical and degenerate NBS1 are different. This is an important conclusion, but the authors should validate it by examining the dynamics of NBS1 of ABCB11.*

We thank the reviewer for raising this interesting point. Since their common origin, ABCB1 and ABCB11 have diverged considerably in the course of evolution, with an adaptation of the ABCB11 TMDs to the presence of only one hydrolysis competent NBS. The bidirectional cross-talk between TMDs and NBDs makes the TMDs a major determinant for NBD dynamics and these will necessarily be different in ABCB1 and ABCB11. NBD dynamics in the NBS1 mutants would thus be convoluted by the intrinsic differences between ABCB1 and ABCB11. Therefore the attempt to use ABCB11 MD simulations as a control for MD simulations of ABCB1 would in our opinion be prone to fail. The adequate controls for MD simulations of the quadruple mutant of ABCB1 are thus ABCB1 wild type and the ABCB1 single E556M.

*6. Fig.7A: The results indicate that E556M restrict the dynamics of the NBD dimer and the quadruple mutant restores back partially such dynamics. This seems to denote that increased dynamics are needed for function. The authors should comment on that.
For me such results are counter-intuitive compared to the results in Fig.6. As the NBS1 is similarly packed is WT and E556M and more relaxed in the quadruple mutant, I would not expect such enhanced dynamics only for WT. Again, similarly to point5, the NBD/NBD dynamics of ABCB11 should also be examined and compared.*

We improved the interpretation of these results in the manuscript.

In a nutshell, our argument is as follows: enzymes bind the substrate tightly in a pre-hydrolytic state, for which restricted dynamics are characteristic. We see that ATP is tightly bound in wild type ABCB1. Prior to hydrolysis wild type and the E556M mutant adopt a state, in which ATP is tightly bound. This state is characterized by restricted dynamics (Fig 7). As in the E556M mutant the hydrolytic activity is abolished by mutation of glutamate E556, the transporter becomes locked in this pre-hydrolytic state, because only the products of the enzymatic reaction (ADP and phosphate) can efficiently be released. The additional amino acid changes introduced in the quadruple mutant prevent the formation of this pre-catalytic state. Our molecular dynamics data show increased mobility of NBS1 and a more loosely bound ATP, as such high dynamics are inconsistent with a pre-hydrolytic state. We can therefore infer that the quadruple mutant does not get trapped in the ATP occluded pre-catalytic state, because the additional three residues prevent its formation.

We rephrased the results and discussion section to explain the concept in greater detail.

*7. Fig.7B: Here the authors examine the correlation between the* *Qloop1-Signature2 and the Signature1-Signature 2 distance. The results for WT and E556M coincide more than those between the WT and the functional quadruple mutant. I would expect that ABCB11 would have a similar correlation with the quadruple mutant, indicative of a different mode of action for degenerate NBSs; and for this is should be examined.*

The distances provide a measure of the NBS and the overall NBD-NBD geometry, and are consistent with the conclusions presented in the manuscript. The question of similarity and difference in conformation and dynamics between ABCB1 and ABCB11 is interesting, but outside the scope of the current manuscript. This manuscript reports on the effect of introducing four residues (as present in ABCB11) into ABCB1.

The proposed comparison between ABCB1 and ABCB11 would report on a different, but closely related question, and would require extensive simulations of both transporters, its mutants and experimental characterization of all findings. Much of the overall transporter dynamics will come from thelarger TMDs, which by conformational cross-talk affects the NBDs. The evolution has let to the divergence of ABCB1 and ABCB11 sequence and consequently of their dynamics. These differences represent confounding element that would convolute withchanges induced by the mutations studied in this manuscript, hampering its interpretation within the scope of the current manuscript.

*8. Fig8: Here the results indicate that the distance between the Q loops are similar for WT and the Quadruple mutant; while the dynamics of the D loops are more similar between WT and the E556M derivative. From my points 5-8 it becomes apparent that the authors should try to relate the properties of the NBD dimer that are critical for function (thus the same for WT and the quadruple derivative) and uncouple them from those that describe probably different dynamics occurring in NBD dimers harboring degenerate sites. As mentioned, for this to be accomplished authors should investigate
1. The corresponding dynamics in ABCB11
2. The effect of critical mutations that affect function and presumably dynamics, alike the double mutant Q475A/Q118A the authors mention in line 305 would be very helpful to be investigated.*

We would like to once more point out the major objective of our experiments. ABCB1 is a full-length transporter with two hydrolysis-competent nucleotide binding sites. There is broad agreement that mutation of glutamate E556 leads to conformational arrest and transport deficiency. In contrast, ABCB11 is a full-length transporter with a non-canonical NBS1, which is not capable of hydrolyzing ATP. The NBD/NBD interface, which is responsible for the formation of the two composite ATP-binding sites, differs between ABCB1 and ABCB11 in only four residues, all located in NBS1. The scientific question was: Can we rescue ABCB1 that has a mutated E556, when changing additional residues. The answer to this question is “yes”. MD-simulations of the full-length transporter as well as biochemical experiments show that conformational dynamics of the transporter are reduced upon introduction of the E556M mutation, and are regained upon introduction of the additional three mutations that are found in analogous position in the hydrolysis incompetent NBS1 of ABCB11. In no way did we attempt to study ABCB11. The major finding is that ABCB1 is released from a conformationally arrested state by an ABCB11 like NBS1. Doing extensive studies of additional mutations in the NBDs of ABCB1 or other ABCB subfamily members, though very interesting in a different context, is beyond the scope of the present manuscript.

The proposed simulations of ABCB11 would be of limited help in answering the posed question of which residues and which properties are critical for the function of ABCB1. Simulations ofABCB11 will show non-identical dynamics and conformations, and it will remain unclear, if data will be the result of the introduced mutations or other differences in sequence differences between ABCB1 and ABCB11.

*9. The authors claim multiple times within the paper, that E556M mutation is deficient in ATP hydrolysis: line 374, 67, 91,47 etc, implying that E556 acts as the catalytic carboxylate to cleave the bond between the beta and gamma phosphate. Thus, authors conclude that in the E556M (and consequently in the degenerate NBSs) ATP is not hydrolyzed and either remains bound to keep the NBDs in the close proximity (responsible for the locked, non-functional conformation in the E556M derivative) or ATP unbinds (lines 398-401). This is contrary to previous published data, that indicate that ATP hydrolysis is achieved but when E556 is mutated, defects are encountered in multiple ATP hydrolysis rounds (Biochemistry 2002,41,13989-14000 Sauna et al & JBC, 2004,279, 31212-31220 Tombline et al). The authors did not proved that hydrolysis is abolished and to my knowledge there is zero evidence in the literature that E556 is the catalytic residue in ABCB1. In line with this, there are controversial results in the literature on the role of the conserved E of Walker B motif (nicely summarized of page 328 of the review Davidson, Chen et al; 2008, doi:10.1128/MMBR.00031-07) .The fact that E556 is not the catalytic residue, seems to be consistent with the findings of the authors, given the fact that it does not contact the beta/gamma phosphate during their simulations (lines 255-263). The authors should comment on this very important point in the manuscript. According to their simulations, what is their role of E556?. As the role of that residue -the main player in their study-, is controversial, they should refer extensively in the introduction about the function of the Walker B motif E residue, and also adapt all their discussion statements and conclusions with respect to the correct role of E556.*

Since the first report by the group of Alan Senior in the 90s showing that mutation of either glutamate in the Walker B motif abolishes transport activity of ABCB1, there has been unanimous agreement that mutation of residue E556 is not compatible with transport.

We agree with the reviewer in that the exact role of E556 in the ATP hydrolysis reaction remains disputed. In the literature, the corresponding residue is typically referred to as the “catalytic” glutamate. Higher resolution structures of ABC transporters have been obtained in the presence of ATP only when the catalytic glutamate E556 is mutated. This supports the notion that the transporter attains a conformationally restrained state and that ATP hydrolysis proceeds only at a low rate. However, it has been shown that the E556Q mutant has very low, but detectable residual ATPase activity in detergent purified ABCB1 (Tombline et al. JBC, 279, 31212-31220, 2004). This low ATPase activity is not sufficient to sustain any detectable transport. In our hands, the steady-state ATPase activity of the E556M mutant does not show any statistically significant difference from controls (Fig. 4B).

The nature of the chemical reaction in the NBSs is still debated (extensively discussed in our recent review by Szöllösi et al,BBABiomembranes, 1860, 818-832, 2018, DOI: 10.1016/j.bbamem.2017.10.028). As shown for many enzymes, there can be multiple ways to abolish their function. All currently proposed mechanisms point to a prominent role for residue E556, in line with pronounced effects that its mutation has on catalytic rates in basically all investigated ABC transporters. A direct contact between the catalytic glutamate E556 and the phosphates is not postulated in all models, as a nucleophilic attack on the phosphate atoms leading to bond breakage between the beta and gamma-phosphate of ATP could also be mediated by an activated OH^-^ nucleophile generated with the contribution from residue E556.

Our results deepen the understanding of the E556 function by adding contextual information provided by the NBS1 interface. We now show that engineering an ABCB11 like NBS1 into ABCB1 results in an active transporter even when the catalytic glutamate is missing. We show that: (i) the E556M mutation abolishes ATPase and transport activity by restraining conformational dynamics of the transporter; (ii) introduction of three additional mutations, as found in the ABCB11 NBS1 restores conformational dynamics, ATPase and transport activity. This is shown in parallel both in biochemical wet lab experiments and by molecular dynamics simulations in silico. We extended the revised manuscript to discuss the role of E566 in catalysis in the discussion.

*Minor Issues:
1. For clarity, I strongly believe that authors should keep the orientation of the NBD dimer identical throughout the paper (Fig.1, Fig.6C, Fig.7D,E, Fig.8C/D). They should create a supplementary figure having multiple orientations of the NBD dimer, indicating with arrows the rotations to go from one to the other. This should only be a ribbon representation in which all
residues relevant for this study and important regions (WalkerA,B; signature motifs, etc) are indicated.*

Upon suggestion of the reviewer we added an additional figure in the supplement (Fig S1) showing the NBDs in ribbon representation and with the important motifs highlighted. However, we are inclined to keep the orientations shown in the original figures, because we believe that it would be impossible to convey the crucial information of Figs 1 and 6 to 8 by keeping the same orientation. For example, the surface of the NBD interface shown in Fig1 and the cross section through the electrostatic field (Fig 7) are orthogonal to each other.

*2. Authors claim that the difference between the NBD dimeric interface between ABCB1 and ABCB11 is limited to 4 residues (line 126). Authors should provide a supplementary table in which all interface interactions in the 2 proteins are indicated*

We added Fig S2 to the supporting information. The figure shows separately for both NBDs all residues of one NBD that are in direct contact with the second NBD across the NBD interface. In addition, contacts are presented in a contact matrix, in which mutated residues and their direct contacts are indicated.

*3. The alignments to conclude that the 4 differing residues between ABCB11 and ABCB1 are conserved in ABCB11 should be placed in the Supplement*

The alignments of ABCB1 and ABCB11 sequence fill close to 100 pages. We would therefore like to refrain from adding them to the supporting information. The procedure used for generating the alignment is described in this manuscript, which can therefore easily be reproduced by our readers.

*Reviewer #2: In this manuscript, Goda et al use various experimental and computation techniques to show that while mutating E556 in one of the nucleotide binding sites abolishes both the catalytic and transport activities of ABCB1 (as previously known), employing three more mutations in the NBD interface based on the ABCB11 sequence will result in the gain of both catalytic and transport activities (at least for some substrates). The experiments and arguments are generally straightforward and the manuscript reports very valuable results; however, there are some issues that need to be addressed.*

*Major points:*

*1) Convergence of simulation data: The computational side of this manuscript could have played a very important role in elucidating the important experimental observations made. However, there is a major issue with the simulation data. The simulations are only 400 ns long and although it might be computationally difficult to perform longer simulations, it is difficult to trust the data if a convergence has not been achieved. Some of the time series provided question the reach of convergence. For instance, the bottom panels of Fig. 6B and Fig. 8B show some important events happening towards the end of the simulation. It is very reasonable to assume that if one continues these simulations for another 400 ns or so we may observe significant shifts in various distributions shown in different plots. It is thus important to continue these simulations until some relative convergence is reached.*

We thank the reviewer for this suggestion. We extended all simulations to 1µs, which required significant computing time. The revised manuscript reports on results that are based on much longer simulations, while remaining in agreement with the results obtained in shorter runs.

*2) Transport activity of the quadruple mutant: The authors state that (lines 238-239) the quadruple mutant was able to limit Hoechst 33342 and calcein accumulation, though not as efficiently as wild-type ABCB1. However, Fig. 5 shows that the calcein transport activity of the quadruple mutant is much closer to that of the E556M mutant than the wild-type ABCB1. I think it is more accurate to state that the gain of transport function is substrate dependent. It is possible that a higher concentration of calcein could result in a better distinction between the transport activity of the single and quadruple mutant but we have not been provided by such data.*

We thank the reviewer for this comment. We carried out additional experiments to expand the information shown in Fig 5. The experiments performed with extended substrate concentration ranges show similar bell-shaped curves for wild-type and the quadruple mutant. These biphasic curves are a hallmark for many ABCB1 substrates, including rhodamine 123, Hoechst 33324 and calcein-AM. The ratios between the transport activity factors of wild type and quadruple mutant are comparable for both Hoechst 33324 and calcein-AM. Therefore we conclude that the transport characteristics for the substrates Hoechst 33324 and calcein-AM are comparable.

*Minor point:

In the Discussion section, it is mentioned that ATP hydrolysis by the NBDs converts the chemical energy stored in the phosphate bonds of ATP into NBD motions. This statement somewhat implies it is the heat released from the hydrolysis that results in the NBD dissociation but even in the absence of this heat, e.g., when the ATPs are not present at all, one expects the equilibrium to shift towards the dissociated NBDs. It is the free energy rather than the heat/enthalpy that matters here.*

We thank the reviewer for pointing this out. We have recently shown that the free energy is the driving force for nucleotide-mediated motions of the NBDs (Szöllősi et al, SciReports 2020, 10(1):2589. doi: 10.1038/s41598-020-59403-7). We modified the manuscript accordingly.
